# Gnpat does not play an essential role in systemic iron homeostasis in murine model

**DOI:** 10.1111/jcmm.15068

**Published:** 2020-02-28

**Authors:** Peng An, Jiaming Wang, Hao Wang, Li Jiang, Jia Wang, Junxia Min, Fudi Wang

**Affiliations:** ^1^ Beijing Advanced Innovation Center for Food Nutrition and Human Health China Agricultural University Beijing China; ^2^ School of Public Health The First Affiliated Hospital Institute of Translational Medicine Collaborative Innovation Center for Diagnosis and Treatment of Infectious Diseases School of Medicine Zhejiang University Hangzhou China; ^3^ Precision Nutrition Innovation Center School of Public Health Zhengzhou University Zhengzhou China

**Keywords:** GNPAT, haemochromatosis, hepcidin, HFE, iron

## Abstract

The *GNPAT* variant rs11558492 (p.D519G) was identified as a novel genetic factor that modifies the iron‐overload phenotype in homozygous carriers of the HFE p.C282Y variant. However, the reported effects of the GNPAT p.D519G variant vary among study populations. Here, we investigated the role of GNPAT in iron metabolism using *Gnpat*‐knockout (*Gnpat^−/−^*), *Gnpat*/*Hfe* double‐knockout (*Gnpat^−/−^Hfe^−/−^* or DKO) mice and hepatocyte‐specific *Gnpat*‐knockout mice (*Gnpat^fl/fl^;Alb‐Cre*). Our analysis revealed no significant difference between wild‐type (*Gnpat^+/+^*) and *Gnpat^−/−^* mice, between *Hfe^−/−^* and DKO mice, or between *Gnpat^fl/fl^ and Gnpat^fl/fl^;Alb‐Cre* with respect to serum iron and tissue iron. In addition, the expression of hepcidin was not affected by deleting *Gnpat* expression in the presence or absence of *Hfe.* Feeding *Gnpat^−/−^* and DKO mice a high‐iron diet had no effect on tissue iron levels compared with wild‐type and *Hfe^−/−^* mice, respectively. *Gnpat* knockdown in primary hepatocytes from wild‐type or *Hfe^−/−^* mice did not alter hepcidin expression, but it repressed BMP6‐induced hepcidin expression. Taken together, these results support the hypothesis that deleting *Gnpat* expression has no effect on either systemic iron metabolism or the iron‐overload phenotype that develops in *Hfe^−/−^* mice, suggesting that GNPAT does not directly mediate iron homeostasis under normal or high‐iron dietary conditions.

## 1 INTRODUCTION

Hereditary haemochromatosis (HH) is an iron disorder characterized by excessive iron absorption from the intestine and the progressive deposition of iron in multiple organs.^1^, ^2^ HH is caused by mutations in the genes that encode the human haemochromatosis protein (*HFE*), transferrin receptor 2 (*TFR2*), haemojuvelin (*HJV*, also known as *HFE2*) and hepcidin (*HAMP*); all of these mutations disrupt the iron‐regulating hormone hepcidin and the downstream iron exporter ferroportin.^1^ The clinical iron‐overload phenotype associated with HH is highly variable,^3^ giving rise to the long‐standing question of whether a genetic modifier underlies this phenotypic heterogeneity.^4^, ^5^, ^6^, ^7^, ^8^, ^9^, ^10^, ^11^, ^12^, ^13^, ^14^, ^15^, ^16^, ^17^, ^18^

Mutations in the *HFE* gene account for the most common form of haemochromatosis; moreover, approximately 1 in 200 Caucasians are carriers of the HFE p.C282Y variant.^19^, ^20^ Given the low penetrance of this variant, several groups have attempted to identify additional genetic loci^21^ that affect iron status both in the general population^10^, ^11^, ^12^ and among homozygous carriers of the HFE p.C282Y variant.^13^, ^15^ Using exome sequencing, McLaren et al^22^ recently found that the prevalence of the p.D519G variant in GNPAT (glyceronephosphate O‐acyltransferase) is higher among males who are homozygous carriers of the HFE p.C282Y variant and present with a severe iron‐overload phenotype, suggesting that *GNPAT* might serve as a genetic modifier of the iron‐overload phenotype in these individuals. Interestingly, even among healthy individuals who do not carry the HFE p.C282Y variant, an association has been found between the GNPAT p.D519G variant and changes in several iron parameters.^23^, ^24^ Nevertheless, the precise role of the GNPAT p.D519G variant in homozygous carriers of the HFE p.C282Y variant is highly controversial.^25^, ^26^, ^27^, ^28^, ^29^, ^30^

*GNPAT* encodes glyceronephosphate O‐acyltransferase, the first enzyme in the ether lipid biosynthesis pathway. In humans, mutations in *GNPAT* have been associated with rhizomelic chondrodysplasia punctata, a condition characterized by severely impaired endochondral bone formation, rhizomelic shortening of the femur and humerus, vertebral disorders, dwarfism, cataract, cutaneous lesions, facial dysmorphism and severe mental retardation with spasticity.^31^ Consistent with the important role of GNPAT in development, *Gnpat*‐deficient mice have growth deficits, male infertility, structural abnormalities in the cerebellum, and impaired myelination and Schwann cell development.^32^, ^33^ Recently, *Gnpat*‐knockout mice were also used to demonstrate that Gnpat plays a protective role in muscle strength^33^ and both hepatic steatosis and steatohepatitis.^34^

Despite epidemiological evidence supporting a role of Gnpat in *HFE*‐linked haemochromatosis, its functional role in regulating iron has not been investigated in vivo. To develop a model for functionally characterizing the role of GNPAT in iron metabolism, we generated *Gnpat*‐knockout (*Gnpat^−/−^*) mice and *Gnpat/Hfe* double‐knockout mice (*Gnpat^−/−^Hfe^−/−^*, hereafter referred to as simply *DKO* mice).

## 2 MATERIALS AND METHODS

### 2.1 Mice

**Figure 1:**
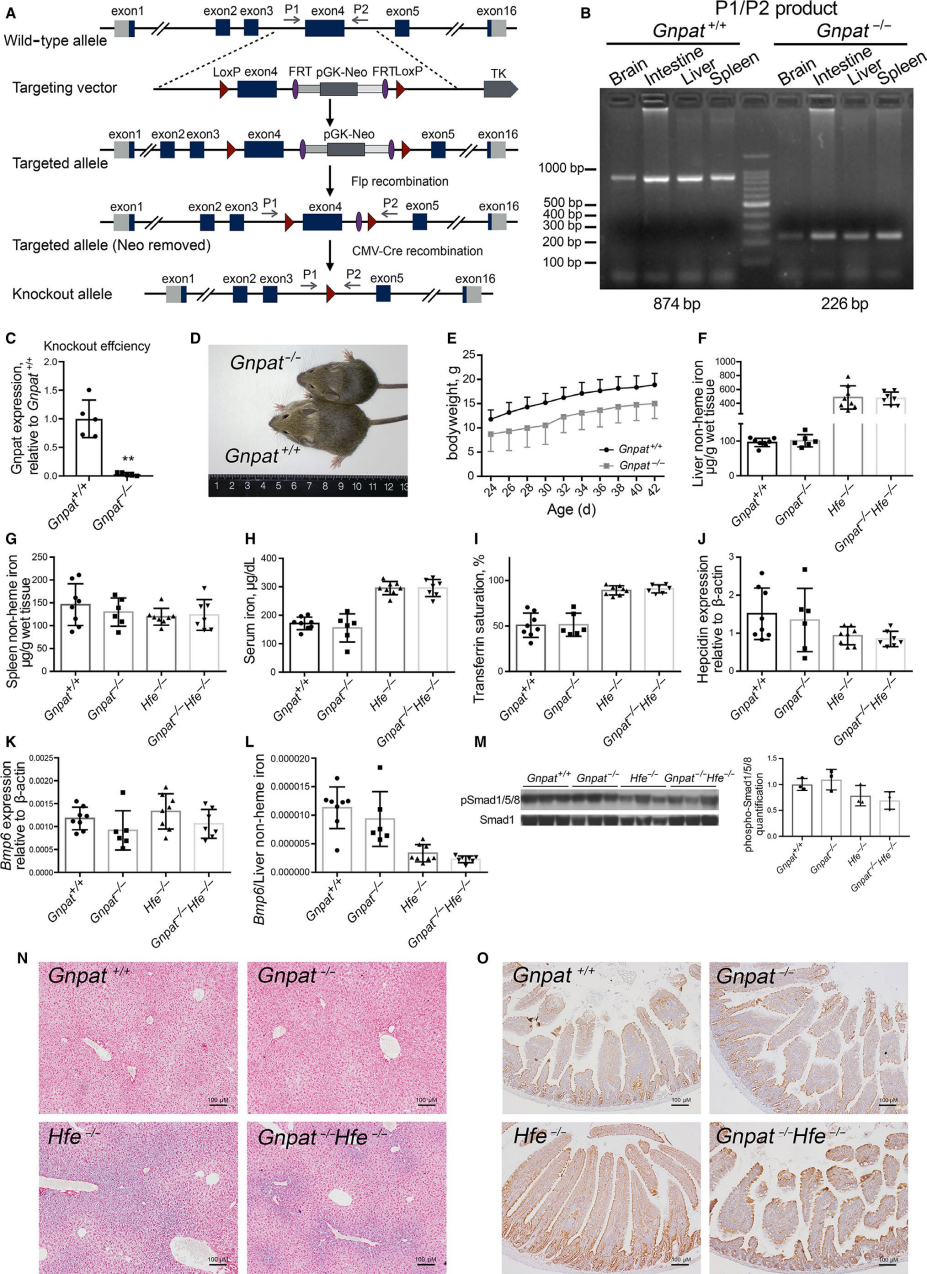
Generation and characterization of *Gnpat^−/−^* mice. A, The targeting strategy for knocking out the mouse *Gnpat* gene. Two LoxP sites flanking exon 4 were inserted in the *Gnpat* gene. Mice carrying the targeted allele were then crossed with CMV‐Cre transgenic mice to generate *Gnpat^+/−^* offspring, which were crossed to produce *Gnpat^+/+^* (wild‐type), *Gnpat^+/−^* and *Gnpat^−/−^* offspring. P1 and P2 indicate the primers that were used to genotype the offspring. B, Genotyping results using primers P1 and P2, which span exon 4, confirm deletion of exon 4 in the *Gnpat* gene in the brain, intestine, liver and spleen of homozygous *Gnpat^−/−^* mice. The predicted sizes of the PCR products for the targeted and knockout alleles are 874 and 226 bp, respectively. C, Knockout efficiency of *Gnpat^−/−^* mice (n = 5). D, A 4‐week‐old female *Gnpat^−/−^* mouse shown next to a female *Gnpat^+/+^* littermate. E, Bodyweight of female *Gnpat^+/+^* (n = 17), *Gnpat^−/−^* (n = 12) versus age. F‐L, Summary of hepatic non‐heme iron (F), splenic non‐heme iron (G), serum iron concentration (H), transferrin saturation (I), hepatic *Hamp1* mRNA level (J), hepatic *Bmp6* mRNA level (K), *Bmp6/*liver non‐heme iron (L) and hepatic phospho‐Smad1/5/8 levels (M) in 6‐week‐old *Gnpat^−/−^*, *Gnpat^+/+^*, *Hfe^−/−^* and *Gnpat^−/−^Hfe^−/−^* mice (n = 6‐8 female mice/group). N, Example images of liver sections obtained from the indicted mice and stained with Perls’ Prussian blue. O, Example images of small intestine sections obtained from the indicated mice and immunostained for ferroportin. The scale bars represent 100 μm. **, *P* < .01 (quantification of immunostained ferroportin of small intestine sections is shown in Figure S1)

**Figure 2:**
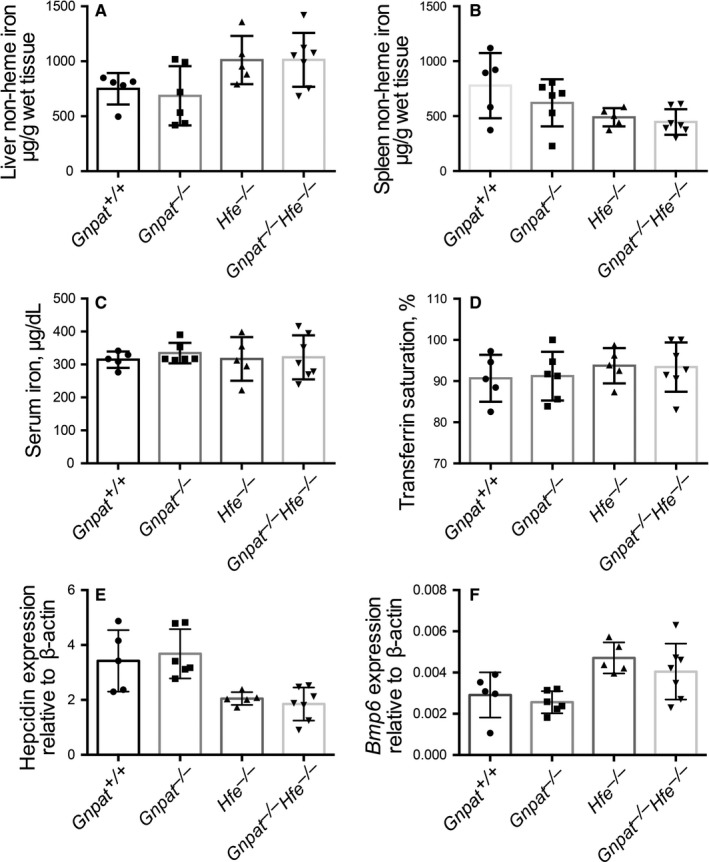
Loss of *Gnpat* expression does not affect iron metabolism under high dietary iron condition. A‐F, At 6 weeks of age, the indicated groups of mice were fed a high‐iron diet for 10 d, and hepatic non‐heme iron (A), splenic non‐heme iron (B), serum iron concentration (C), transferrin saturation (D), hepatic *Hamp1* mRNA (E) and hepatic *Bmp6* mRNA (F) were measured (n = 5‐7 female mice per group)

Global *Gnpat*‐knockout (*Gnpat^−/−^*) mice were generated at Shanghai Biomodel Organism Science & Technology Development Co., Ltd. using the Cre‐LoxP recombination approach (see Figure 1A). In brief, two LoxP sites flanking exon 4 in the *Gnpat* gene were introduced into embryonic stem cells derived from C57BL/6J129S3 mice. Chimeric mice were obtained by injecting targeted embryonic stem cells into C57BL/6 blastocysts. Chimeric mice containing the *Gnpat‐flox* allele were crossed with C57BL/6J mice to obtain *Gnpat‐flox* mice. Mice carrying the *Gnpat‐flox* allele were then crossed with CMV‐Cre transgenic mice^35^ on a C57BL/6J background to generate *Gnpat^−/−^* mice. *Hfe^−/−^* mice were kindly provided by Dr Nancy C. Andrews^36^ and were maintained on the 129/SvEvTac background. To obtain *Gnpat^−/−^Hfe^−/−^* double‐knockout (DKO) mice, we crossed *Gnpat^−/−^* mice with *Hfe^−/−^* mice, obtaining offspring with a mixed genetic background. *Gnpat‐flox* mice were crossed with albumin‐Cre (*Alb‐Cre*) transgenic mice (129/SvEvTac background) to obtain hepatocyte‐specific *Gnpat*‐knockout mice (*Gnpat^fl/fl^;Alb‐Cre*). The mice used in this study were 6‐week‐old (*Gnpat^−/−^*, *Hfe^−/−^* or DKO) or 8‐week‐old (*Gnpat^fl/fl^;Alb‐Cre*) littermates on a mixed background of C57BL/6J and 129/SvEvTac (*Gnpat^−/−^*, *Hfe^−/−^*, DKO and *Gnpat^fl/fl^;Alb‐Cre*). All mice were maintained under specific pathogen‐free conditions and—except where indicated otherwise—fed AIN‐76A standard rodent diet containing 50 mg iron/kg (Research Diets, Inc). The high‐iron diet used for the experiments in Figure 2 was the same rodent diet AIN‐76A containing 8.3 g/kg carbonyl iron (Research Diets, Inc). All experimental protocols were approved by the Institutional Animal Care and Use Committee of the Laboratory Animal Center, Zhejiang University.

### 2.2 Serum iron, tissue non‐heme iron and haematology parameters

Tissue non‐heme iron was measured as previously described.^37^ To measure serum iron and transferrin saturation, whole blood was collected by heart puncture and allowed to coagulate at room temperature for 2 hours; serum was then obtained by centrifugation at 2400 *g*. Serum iron (SI) concentration and unsaturated iron‐binding capacity (UIBC) were measured using a colorimetry‐based assay (Pointe Scientific) in accordance with the manufacturer's instructions. Total iron‐binding capacity (TIBC) and transferrin saturation (TS) were calculated using the following equations: TIBC = SI + UIBC and TS = SI/TIBC*100.

### 2.3 Ferroportin immunohistochemistry and Perls’ iron staining

Immunohistochemical detection of intestinal ferroportin and Perls’ Prussian blue iron staining were performed as previously described.^38^ The protein expression levels of ferroportin are quantified using ImageJ software.

### 2.4 Plasmids and cell culture

The wild‐type *GNPAT* cDNA (Ensembl transcript ID: ENST00000366647.8) was cloned from the HepG2 cell line. The *GNPAT* cDNA encoding the p.D519G mutation was generated by site‐directed mutagenesis. Both the wild‐type and p.D519G *GNPAT* cDNAs were cloned into the pmCherry vector. The sequences of both constructs were confirmed by sequencing. For transfection experiments, HepG2 and Huh‐7 cells were cultured in 6‐well plates at 37°C in humidified air containing 5% CO_2_. The culture medium (Dulbecco's modified Eagle's medium containing 4.5 g/L glucose) was supplemented with 10% fetal calf serum and 1% penicillin‐streptomycin. The cells were cultured for 24 hours, mock‐transfected or transfected with 10 ng/mL BMP4 (as a positive control), wild‐type GNPAT, or p.D519G GNPAT using Lipofectamine 3000 (Thermo Fisher Scientific), then cultured for an additional 36 hours.

### 2.5 siRNA transfection

HepG2 cells were plated in 12‐well plates and cultured at 37°C in 5% CO_2_ with Dulbecco's modified Eagle's medium (Gibco) containing 10% heat‐inactivated fetal bovine serum (Gibco) (v/v). The cells were then transfected with siRNA targeting human *GNPAT*/mouse *Gnpat* gene or control non‐specific siRNA (30 pmol/well; TransSheep; siRNA sequences were listed in Table S2) at 70% confluence with Lipofectamine 3000 (Invitrogen). Twenty‐four hours after transfection, human recombinant BMP6 (R&D systems) was added to a final concentration of 20 ng/mL and cells were incubated for additional 12 hours. The cells were then collected for RNA extraction and quantitative real‐time PCR detection. Isolation of mouse primary hepatocytes was performed as previously described. 39 The isolated primary hepatocytes from either wild‐type or *Hfe^−/−^* mice were cultured in Dulbecco's modified Eagle's medium containing 10% fetal bovine serum. The siRNA transfection and BMP6 treatment steps are comparable with HepG2 cells.

### 2.6 RNA extraction and quantitative real‐time PCR

RNA was extracted from cells and liver tissues and analysed using quantitative real‐time PCR analysis as previously described.^40^

### 2.7 Western blot analysis

Hepatic tissue was lysed using RIPA lysis buffer, and total protein (40 µg/sample) was loaded on a 10% sodium dodecyl sulphate polyacrylamide gel and separated by electrophoresis. After transferring the proteins to a membrane, the following primary antibodies were used for Western blot analysis: rabbit anti‐phospho‐Smad1/5/8 (Cell Signaling Technology), rabbit anti‐Smad1 (Cell Signaling Technology) and mouse anti‐β‐actin (Sigma‐Aldrich).

### 2.8 Statistical analysis

All summary data are presented as the mean ± SD. Where indicated, groups were compared using the analysis of variance (ANOVA) and Tukey's test for multiple comparisons. All statistical analyses were performed using GraphPad Prism 8, and differences with a *P*‐value < .05 were considered significant.

## 3 RESULTS

### 3.1 Association between GNPAT p.D519G and iron‐overload phenotype in homozygous carriers of the HFE p.C282Y variant in human populations

We summarized published population studies on *GNPAT* p.D519G variant in Table S1. Following the first report of the increased prevalence of the GNPAT p.D519G variant among homozygous carriers of HFE p.C282Y by McLaren et al,^22^ an additional nine studies were published.^23^, ^24^, ^25^, ^26^, ^27^, ^28^, ^29^, ^30^, ^41^ The evidence obtained from human population studies with respect to the role of the GNPAT p.D519G variant remains controversial. Therefore, an open question is whether the GNPAT p.D519G variant plays a direct role in iron metabolism or whether this variant functions in regulating HFE‐linked haemochromatosis.

### 3.2 Hepcidin expression and tissue iron levels are normal in *Gnpat^−/−^* or *Gnpat*/*Hfe* double‐knockout (*Gnpat^−/−^Hfe^−/−^*) mice

To investigate the role of GNPAT in iron metabolism in a controlled genetic system, we generated a *Gnpat‐*knockout (*Gnpat^−/−^*) mouse (Figure 1A‐C). Consistent with previous reports,^32^, ^33^ our *Gnpat^−/−^* mice are smaller in size (Figure 1D) compared with wild‐type (*Gnpat^+/+^*) littermates. Moreover, *Gnpat^−/−^* mice have an increased rate of early mortality. Nearly, all male *Gnpat^−/−^* mice cannot survive longer than 4 weeks, and most of them suddenly died around 2 weeks old, comparable with previous reports.^32^ The estimated survival rate of 6‐week‐old *Gnpat^−/−^* mice is <40%.

We then investigated the effects of deleting *Gnpat* in *Hfe*‐knockout (*Hfe^−/−^*) mice, which develop a phenotype similar to HFE‐linked haemochromatosis in human. We therefore generated *Gnpat*/*Hfe* double‐knockout mice (referred to hereafter as DKO mice) by crossing our *Gnpat^−/−^* mice with *Hfe^−/−^* mice. To our surprise, we found no significant difference between *Gnpat^−/−^* mice and *Gnpat^+/+^* mice or between *Hfe^−/−^* mice and *DKO* mice with respect to hepatic non‐heme iron (Figure 1E), splenic non‐heme iron (Figure 1F), serum iron concentration (Figure 1G) or transferrin saturation (Figure 1H). In addition, we found no significant difference in hepatic *Hamp1* mRNA (Figure 1I), hepatic *Bmp6* mRNA (Figure 1J), the *Bmp6*/liver non‐heme iron ratio (Figure 1K) or hepatic phospho‐Smad1/5/8 levels (Figure 1L and M). Finally, deleting *Gnpat* had no effect on iron distribution in the liver (Figure 1N) or ferroportin protein in the small intestine (Figure 1O, the ferroportin expression was quantified in Figure S1). Taken together, these results indicate that deleting *Gnpat* does not significantly affect iron metabolism, nor does it affect the iron‐overload phenotype in *Hfe^−/−^* mice.

### 3.3 Iron metabolism in both *Gnpat^−/−^*and DKO mice is not affected by high dietary iron

Next, to explore whether Gnpat plays a role in regulating either the absorption of dietary iron or the deposition of iron in the liver, we fed 6‐week‐old *Gnpat^+/+^*, *Gnpat^−/−^*, *Hfe^−/−^* and DKO mice an iron‐rich diet for 10 days. Similar to our results obtained with mice that were fed a standard diet, we found no significant difference between *Gnpat^+/+^* and *Gnpat^−/−^* mice or between *Hfe^−/−^* and *Gnpat^−/−^Hfe^−/−^* mice with respect to hepatic non‐heme iron (Figure 2A), splenic non‐heme iron (Figure 2B), serum iron concentration (Figure 2C), transferrin saturation (Figure 2C), hepatic *Hamp1* mRNA (Figure 2E) or hepatic *Bmp6* mRNA (Figure 2F). Taken together, these results indicate that GNPAT does not play a key role in iron metabolism under high dietary iron.

### 3.4 Hepcidin expression and tissue iron levels are normal in hepatocyte‐specific *Gnpat*‐knockout (*Gnpat^fl/fl^;Alb‐Cre*) mice

**Figure 3:**
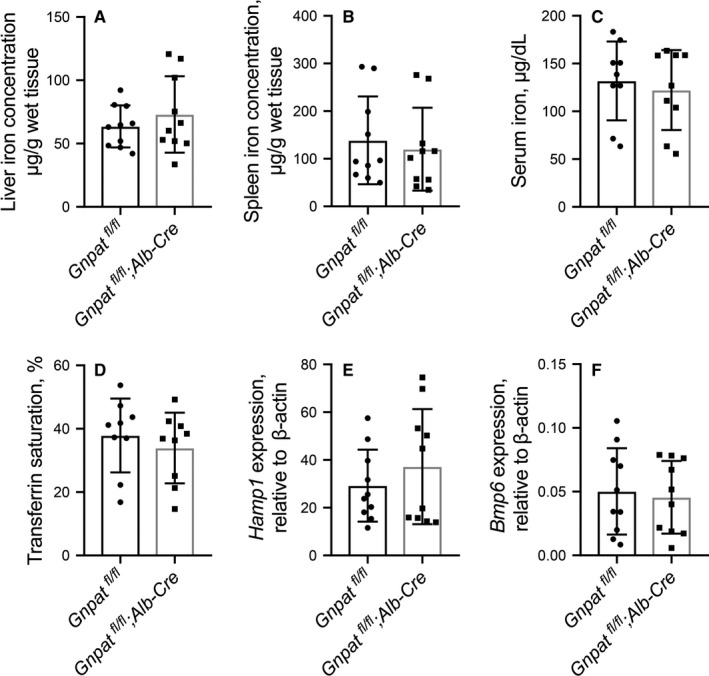
Hepatocyte‐specific ablation of *Gnpat* (*Gnpat^fl/fl^;Alb‐Cre*) does not alter iron metabolism. Summary of hepatic non‐heme iron (A), splenic non‐heme iron (B), serum iron concentration (C), transferrin saturation (D), hepatic *Hamp1* mRNA (E) and hepatic *Bmp6* mRNA (F) of 8‐week‐old male *Gnpat^fl/fl^* mice (n = 10) and *Gnpat^fl/fl^;Alb‐Cre* mice (n = 10)

To avoid the disturbance of mortality brought by global *Gnpat* ablation in male *Gnpat^−/−^* mice (Figure 1), *Gnpat‐flox* mice were further crossed with albumin‐Cre (*Alb-Cre*) transgenic mice to generate hepatocyte‐specific *Gnpat*‐knockout mice (*Gnpat^fl/fl^;Alb‐Cre*). Hepatocyte‐specific *Gnpat*‐knockout mice developed normally and showed similar bodyweight with wild‐type mice. There was no significant difference between *Gnpat^fl/fl^* and *Gnpat^fl/fl^;Alb‐Cre* male mice in hepatic non‐heme iron (Figure 3A), splenic non‐heme iron (Figure 3B), serum iron concentration (Figure 3C) or transferrin saturation (Figure 3D). The hepatic *Hamp1* mRNA (Figure 3E) and *Bmp6* mRNA (Figure 3F) are comparable between *Gnpat^fl/fl^* mice and *Gnpat^fl/fl^;Alb‐Cre* mice.

### 3.5 Knockdown of human *GNPAT* in HepG2 cells and mouse *Gnpat* in primary hepatocytes

**Figure 4:**
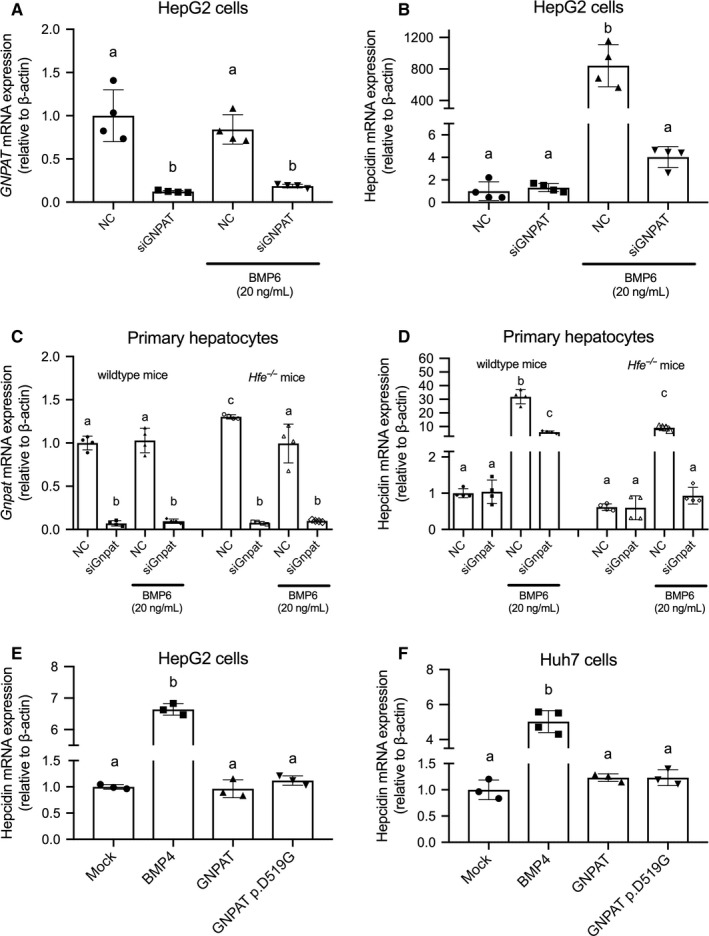
*GNPAT* knockdown in HepG2 cells and primary hepatocytes of wild‐type or *Hfe^−/−^* mice. HepG2 cells were transfected with siRNA targeting human *GNPAT* gene (siGNPAT) or control non‐specific siRNA (NC). *GNPAT* (A) and hepcidin (B) expressions were detected under basal condition or in the presence of 20 ng/mL BMP6. Primary hepatocytes from wild‐type or *Hfe^−/−^* mice were transfected with siRNA targeting mouse *Gnpat* gene (siGnpat) or control non‐specific siRNA (NC). *Gnpat* (C) and hepcidin (D) expressions were detected under basal condition or in the presence of BMP6. HepG2 (E) and Huh‐7 (F) cells were transfected with the indicated constructs, and hepcidin mRNA was measured 36 h after transfection. Groups labelled without a common letter were significantly different (*P* < .05; analysis of variance)

We used small interfering RNA (siRNA) targeting human *GNPAT* in HepG2 cells (Figure 4A). After transfected by siRNA targeting *GNPAT*, no difference was observed in hepcidin expression under basal condition compared with cells transfected with non‐specific control (Figure 4B). Under 20 ng/mL human recombinant BMP6 treatment, significant suppression of BMP6‐induced hepcidin expression can be observed after knocking down *GNPAT* gene (Figure 4B). SiRNA targeting mouse *Gnpat* was also used to transfect primary hepatocytes from wild‐type and *Hfe^−/−^* mice (Figure 4C). Under basal condition, results are consistent with the results from HepG2 cells; we found no significant change in hepcidin expression after knocking down *Gnpat* in the primary hepatocytes of wild‐type or *Hfe^−/−^* mice (Figure 4). The knockdown of *Gnpat* can effectively repress BMP6‐induced hepcidin expression in the presence of 20 ng/mL BMP6 in wild‐type or *Hfe^−/−^* primary hepatocytes (Figure 4D).

### 3.6 Expressing the GNPAT p.D519G variant does not affect hepcidin expression in human cells expressing the HFE p.C282Y variant

Lastly, we examined whether the GNPAT p.D519G variant affects hepcidin expression in two human hepatic cell lines. Both HepG2 cells (Figure 4E) and Huh‐7 cells (Figure 4F) were either mock‐transfected or transfected with wild‐type human GNPAT or the GNPAT p.D519G variant, and hepcidin mRNA was measured. Consistent with our in vivo results, we found that expressing either wild‐type GNPAT or the p.D519G variant had no effect on hepcidin expression; as a positive control, overexpressing BMP4 significantly increased hepcidin mRNA levels in both cell lines.

## 4 DISCUSSION

*GNPAT* encodes glyceronephosphate O‐acyltransferase, the first enzyme in the ether lipid biosynthesis pathway. In humans, mutations in the *GNPAT* gene have been associated with rhizomelic chondrodysplasia punctata, a condition characterized by severely impaired endochondral bone formation, rhizomelic shortening of the femur and humerus, vertebral disorders, dwarfism, cataract, cutaneous lesions, facial dysmorphism and severe mental retardation with spasticity.^31^ Consistent with its important role in development, deleting *Gnpat* expression in mice gives rise to a variety of postnatal defects, including impaired growth. 32, 33 We observed a similar phenotype in our *Gnpat^−/−^* and *Gnpat^−/−^Hfe^−/−^* double‐knockout mice. In addition, and consistent with previous reports,^32^ our male *Gnpat*‐knockout mice have decreased fertility and early mortality (data not shown).

A wide range of global and tissue‐specific knockout mouse models, including *Hfe‐*, *TfR2‐*, *Hjv‐* and *Hamp1*‐knockout mice, have been studied extensively in an effort to understand haemochromatosis and iron homeostasis in humans. Here, we provide evidence that the global deletion of *Gnpat* expression has no effect on iron metabolism, nor does it affect the development of haemochromatosis in *Hfe*‐deficient mice. These in vivo data argue against the hypothesis that GNPAT is a genetic modifier that independently regulates iron metabolism in order to exacerbate the iron‐overload phenotype in patients with HFE‐linked haemochromatosis.

McLaren et al examined the prevalence of the GNPAT p.D519G variant among 22 homozygous carriers of the HFE p.C282Y variant with markedly increased iron stores and found that the GNPAT p.D519G variant was present in 73% of patients in this specific subgroup.^22^ The authors also reported that the frequency of the GNPAT p.D519G allele among male patients with a severe iron phenotype (38.6%) was significantly higher than among Americans of European descent (20.6%); in contrast, none of the males who presented with a mild iron phenotype were carriers of the GNPAT p.D519G allele.^22^ In subsequent studies, this apparent increased prevalence of the GNPAT p.D519G variant was also confirmed in two additional populations.^25^, ^41^ Moreover, the GNPAT p.D519G variant was reported to be associated with increased iron parameters both in homozygous carriers of the HFE p.C282Y variant^41^ and in healthy individuals.^23^, ^24^ Interestingly, however, several other groups found no increase in the prevalence of the GNPAT p.D519G variant in large cohorts of homozygous carriers of the HFE p.C282Y variant,^26^, ^28^, ^29^, ^30^ and no significant association was found between the GNPAT p.D519G variant and serum ferritin levels.^25^, ^26^, ^27^, ^28^, ^29^, ^30^

McLaren et al also found that knocking down *GNPAT* expression in HepG2 cells significantly reduced hepcidin expression but had no effect on phosphorylated Smad1/5/8 level, leading to the hypothesis that GNPAT may regulate iron metabolism via hepcidin independent of the Smad1/5/8 pathway.^22^ In contrast, we found that global deleting *Gnpat* or hepatocyte‐specific deleting *Gnpat* had no significant effect on hepatic hepcidin (Figure 1I and Figure 3E), even under high dietary iron. *GNPAT* knockdown in HepG2 cells or the primary hepatocytes of wild‐type and *Hfe^−/−^* mice did not alter the expression of hepcidin without the presence of exogenous BMP6 (Figure 4B and D). It is interesting that knocking down *GNAPT* prevents exogenous BMP6 from activating hepcidin expression in human cell line and *Hfe^−/−^* mouse primary hepatocytes (Figure 4B and D), whereas *Gnpat*‐ or *Gnpat^−/−^Hfe^−/−^*‐knockout mice have unchanged endogenous hepcidin and BMP6 expression under normal or high‐iron diet condition (Figure 1J and K; Figure 2E and F). This finding is supported by previous reports that *Hfe^−/−^* mice had blunted response to Bmp6 treatment.^42^ Based on our results presented in Figure 4, GNPAT might inhibit hepcidin expression under a relatively high dose of BMP6 treatment. However, under physiological condition, it did not regulate iron‐induced hepcidin expression.

On the other hand, we cannot rule out the possibility that the GNPAT p.D519G variant has another, currently unknown effect when combined with the HFE p.C282Y variant. In this respect, a Gnpat p.D519G knock‐in mouse might provide valuable insight into the role of this variant in iron metabolism. Moreover, given the developmental abnormalities and early mortality among *Gnpat^−/−^* mice—particularly among male mice—long‐term studies of iron accumulation are currently not feasible; therefore, an inducible *Gnpat*‐knockout allele might provide a more suitable model for studying the long‐term time effects of deleting *Gnpat* expression on iron metabolism.

In conclusion, our results indicate that GNPAT does not play an essential role in iron metabolism or in the development of HFE‐linked haemochromatosis in an in vivo mouse model. Moreover, our in vitro experiments using two human hepatic cell lines indicate that the GNPAT p.D519G variant does not affect hepcidin expression.

## CONFLICT OF INTEREST

The authors declare no competing interests.

## AUTHOR CONTRIBUTIONS

FW, JM and PA designed the study. PA, JW (Jiaming Wang), HW, LJ and JW (Jia Wang) performed the in vivo experiments. PA and JW (Jiaming Wang) performed the in vitro experiments. PA and JW (Jiaming Wang) analysed the data. PA, FW and JM wrote the manuscript.

## Data Availability

The data that support the findings of this study are available from the corresponding author upon reasonable request.
