# Supplementary figures and images for: Gnpat does not play an essential role in systemic iron homeostasis in murine model
Source: J Cell Mol Med. 2020 Feb 28;24(7):4118–26. doi: 10.1111/jcmm.15068 (PMC7171407; doi:10.1111/jcmm.15068)

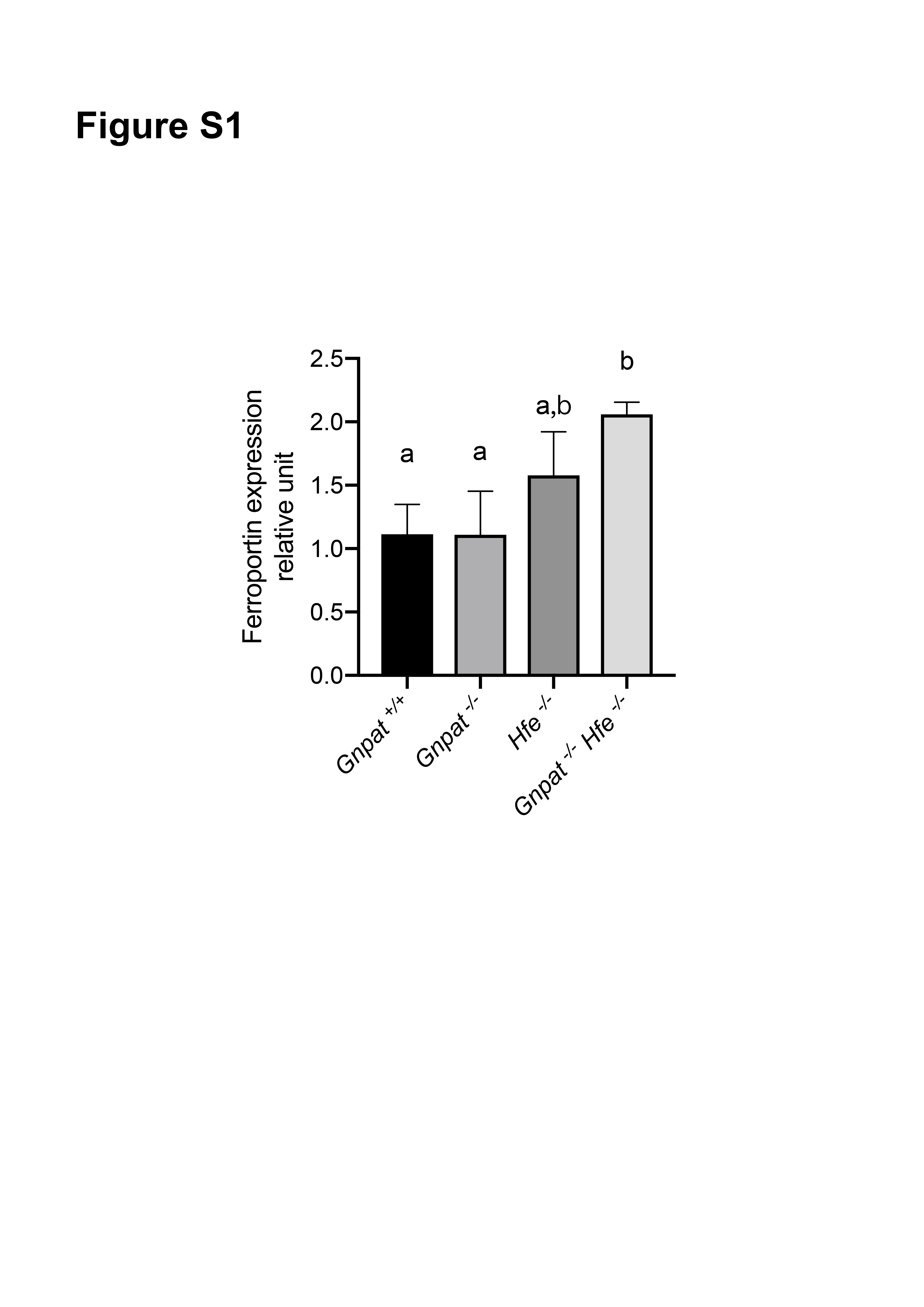

Supplement: Supplementary file 1 [file JCMM-24-4118-s001.tif]

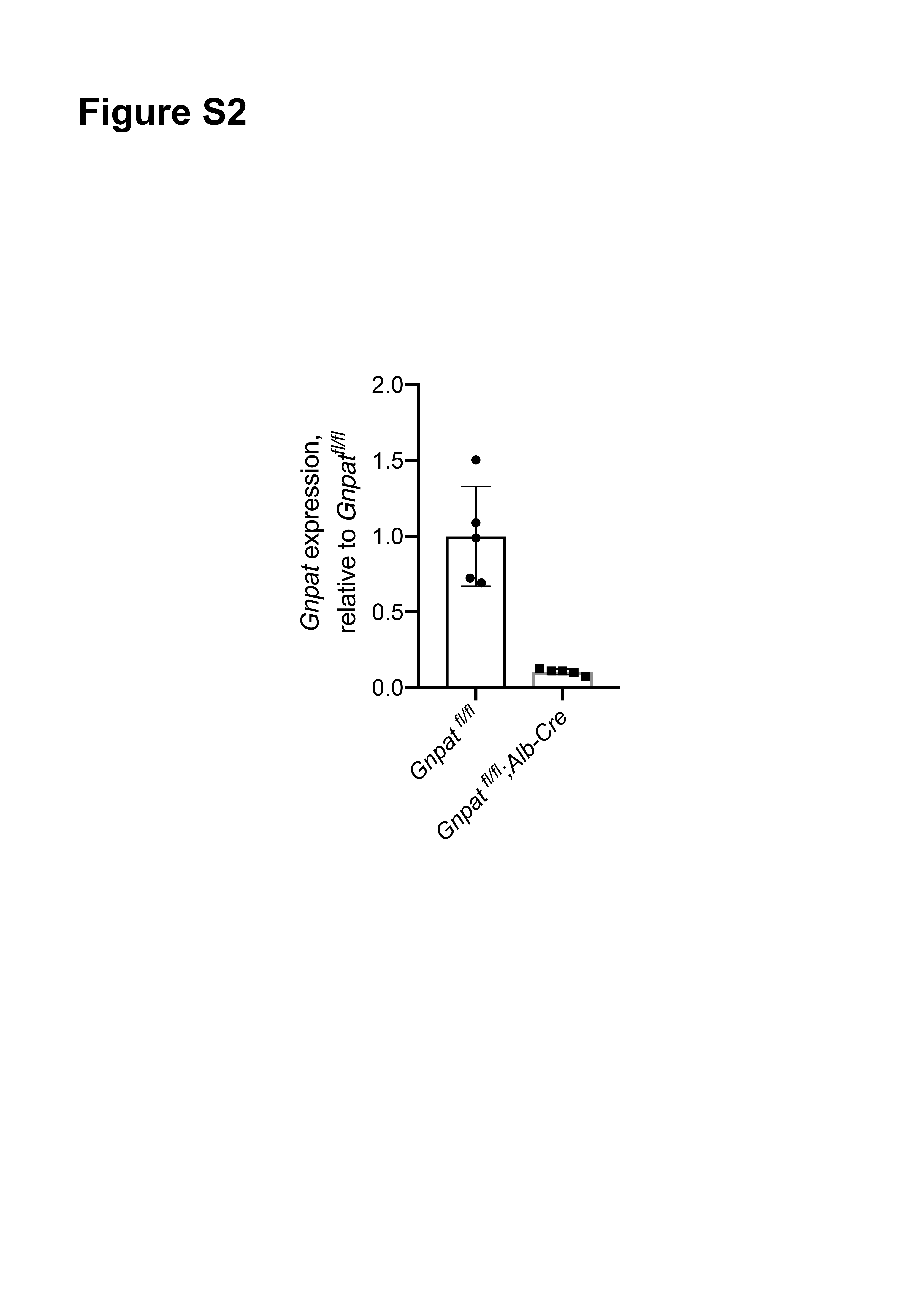

Supplement: Supplementary file 2 [file JCMM-24-4118-s002.tif]
