# Supplementary material for: Gnpat does not play an essential role in systemic iron homeostasis in murine model
Source: J Cell Mol Med. 2020 Feb 28;24(7):4118–26. doi: 10.1111/jcmm.15068 (PMC7171407; doi:10.1111/jcmm.15068)
Supplement: Supplementary file 3 [file JCMM-24-4118-s003.docx]

Table S1. A summary of human population studies of GNPAT p.D519G and iron phenotypes.

| Publication | Population | Results | Support the role of GNPAT p.D519G |
| --- | --- | --- | --- |
| McLaren et al ^1^ | Case: Male HFE p.C282Y homozygotes with markedly increased iron store (n=22)  Control: Male HFE p.C282Y homozygotes with normal or mildly increased iron store (n=13) | GNPAT p.D519G enriched in HFE p.C282Y homozygotes with markedly increased iron store | Yes |
| Besson-Fournier et al ^2^ | HFE p.C282Y homozygous patients (n=284, 174 males, 110 females) | GNPAT p.D519G was not associated with serum ferritin | No |
|  | *Subgroup* |  |  |
|  | Case: Male HFE p.C282Y homozygotes with most severe iron stores (n=87)  Control: European Americans in Exome Variant Server (n=4300) | GNPAT p.D519G enriched in HFE C282Y homozygotes with severe iron store | Yes |
| James et al ^3^ | Case: HFE p.C282Y homozygotes with markedly increased iron store (n=41, 38 males, 3 females)  Control: Male HFE p.C282Y homozygotes with normal or mildly increased iron store (n=15) | The odds of participants with GNPAT p.D519G positivity having markedly increased iron stores were 9.2 times higher (95% CI [1.8, 46.1]) than those of participants without p.D519G. | Yes |
| Hsiao et al ^4^ | 83 healthy female, iron indices before and after iron supplementation | Fasting serum iron, serum iron after iron supplementation, transferrin saturation after iron supplementation were higher in GNPAT p.D519G than in wildtype subjects | Yes |
| Rametta et al ^5^ | 59 individuals (not carrying HFE p.C282Y or p.H63D) participating oral iron tolerance test | GNPAT p.519G carriers had a higher area under the curve of serum iron and transferrin saturation during the oral iron tolerance test | Yes |
| Bardou-Jacquet et al ^6^ | HFE p.C282Y homozygous hemochromatosis patients (n=748, 274 males, 474 females) | GNPAT p.D519G is not associated with iron burden or fibrosis | No |
|  | *Subgroup* |  |  |
|  | Case: Male HFE p.C282Y homozygotes with most severe iron stores (n=274)  Control: European Americans in Exome Variant Server | GNPAT p.D519G enriched in HFE p.C282Y homozygotes with severe iron store | Yes |
| Ryan et al ^7^ | Case: Male HFE p.C282Y homozygotes with serum ferritin >1000 μg/L (n=57)  Control: Male HFE C282Y homozygotes with serum ferritin >1000 μg/L (n=100) | No enrichment of GNPAT p.D519G was found in HFE p.C282Y homozygotes with serum ferritin >1000ug/L | No |
| Levstik et al ^8^ | HFE p.C282Y homozygotes (n=78) | Serum ferritin did not significantly differ among p.C282Y homozygous who were GNPAT p.D519G homozygotes, heterozygotes and wildtype. | No |
| Greni et al ^9^ | Healthy HFE wildtype individuals (n=169) | No significant difference in serum ferritin and liver iron concentration according to the genotypes of GNPAT p.D519G | No |
|  | HFE p.C282Y homozygotes (n=298) |  |  |
|  | male HFE p.C282Y homozygotes (n=205) |  |  |
| Tchernitchko et al ^10^ | HFE p.C282Y homozygous hemochromatosis patients (n=512, 276 males, 236 females) | No significant association of GNPAT p.D519G with serum ferritin and iron removed by phlebotomy | No |
|  | Male HFE p.C282Y homozygotes (n=276) |  |  |

**References**

1. McLaren CE, Emond MJ, Subramaniam VN, et al. Exome sequencing in HFE C282Y homozygous men with extreme phenotypes identifies a GNPAT variant associated with severe iron overload. *Hepatology* 2015; 62; 429–39.

2. Besson-Fournier C, Russell J, Ryan JD, et al. Further support for the association of GNPAT variant rs11558492 with severe iron overload in hemochromatosis. *Hepatology* 2015; 63; 2054–5.

3. James BC, Chen W-P, Emond MJ, et al. GNPAT p.D519G is independently associated with markedly increased iron stores in HFE p.C282Y homozygotes. *Blood Cell Mol Dis* 2017; 63; 15–20.

4. Hsiao S-C, Lee C-T, Pei S-N. GNPATvariant is associated with iron phenotype in healthy Taiwanese women: A population without the HFEC282Y mutation. *Hepatology* 2016; 63; 2057–8.

5. Rametta R, Dongiovanni P, Fargion S, et al. GNPAT p.D519G variant and iron metabolism during oral iron tolerance test. *Hepatology* 2017; 65; 384–5.

6. Bardou-Jacquet E, de Tayrac M, Mosser J, et al. GNPAT variant associated with severe iron overload in HFE hemochromatosis. *Hepatology* 2015; 62; 1917–8.

7. Ryan E, Russell J, Ryan JD, et al. GNPAT variant is not associated with severe iron overload in Irish C282Y homozygotes. *Hepatology* 2016; 63; 2055–6.

8. Levstik A, Stuart A, Adams PC.  GNPAT variant (D519G) is not associated with an elevated serum ferritin or iron removed by phlebotomy in patients referred for C282Y-linked hemochromatosis. *Ann Hepatol* 2016; 15; 907–10.

9. Greni F, Valenti L, Mariani R, et al. GNPAT rs11558492 is not a Major Modifier of Iron Status: Study of Italian Hemochromatosis Patients and Blood Donors. *Ann Hepatol* 2017; 16; 451–6.

10. Tchernitchko D, Scotet V, Lefebvre T, et al. GNPAT polymorphism rs11558492 is not associated with increased severity in a large cohort of HFE p.Cys282Tyr homozygous patients. *Hepatology* 2017; 65; 1069–71.
