# Supplementary material for: Gnpat does not play an essential role in systemic iron homeostasis in murine model
Source: J Cell Mol Med. 2020 Feb 28;24(7):4118–26. doi: 10.1111/jcmm.15068 (PMC7171407; doi:10.1111/jcmm.15068)
Supplement: Supplementary file 4 [file JCMM-24-4118-s004.docx]

Table S2. Primers used for *GNPAT* knockdown experiment.

| **siRNA** | **Sequence** |
| --- | --- |
| human *GNPAT*- forward | GGGCUGUAUUCUCUGAAUATT |
| human *GNPAT*- reverse | UAUUCAGAGAAUACAGCCCTT |
| mouse *Gnpat* - forward | CCAUUCGGUUCUUUGCCUUTT |
| mouse *Gnpat* - reverse | AAGGCAAAGAACCGAAUGGTT |
